# Penile cancer metastasizing to the breast: a case report

**DOI:** 10.1186/s13256-016-0829-3

**Published:** 2016-03-09

**Authors:** Gianluca Franceschini, Alejandro Martin Sanchez, Alba Di Leone, Assunta Scaldaferri, Massimo Ferrucci, Antonio Mulè, Melania Costantini, Riccardo Masetti

**Affiliations:** Multidisciplinary Breast Center, Università Cattolica del Sacro Cuore, Policlinico Agostino Gemelli, Largo Agostino Gemelli, 8, Roma, 00168 Italy

**Keywords:** Breast metastases, Penile cancer, Penile metastases

## Abstract

**Background:**

Penile cancer is a relatively uncommon cancer in developed nations. Metastatic disease is rare, but lymphatic or vascular spreading has been previously reported to the liver, lungs, bones, brain, heart and skin.

**Case presentation:**

We report a case of a 49-year-old white man with a penile squamous cell carcinoma previously treated with partial penectomy and bilateral inguinal lymph node dissection, followed by adjuvant therapy. Three years after treatment, the primitive neoplasm metastasized to the breast, presenting as a painful lump. Differentials of a secondary versus a malignant primary tumor were considered and in view of a diagnostic dilemma the lesion was excised.

**Conclusions:**

This case is unusual in its site of metastatic progression as well as in its pattern of clinical presentation. Awareness of such a condition by physicians is mandatory in order to make an early diagnosis and start prompt and correct therapeutic planning.

## Background

Penile cancer is a rare malignancy in developed nations, with an annual incidence varying from 0.3 to 1 per 100,000 per year, accounting for approximately 0.4 to 0.6 % of all malignancies [1]. Approximately 95 % of penile cancers are squamous cell carcinomas, which easily spread locally through lymphatic or vascular channels [2–4]. Conversely, metastatic disease is rare, primarily disseminating to the liver, lung and bone although brain, dorsal spine, heart, retroperitoneum and skin metastases have been reported [5–10].

We describe a rare case of penile squamous cell carcinoma that metastasized to the breast, resulting in a painful breast lump. To the best of our knowledge this is the first report of breast metastasis from a penile carcinoma.

## Case presentation

**Fig. 1:**
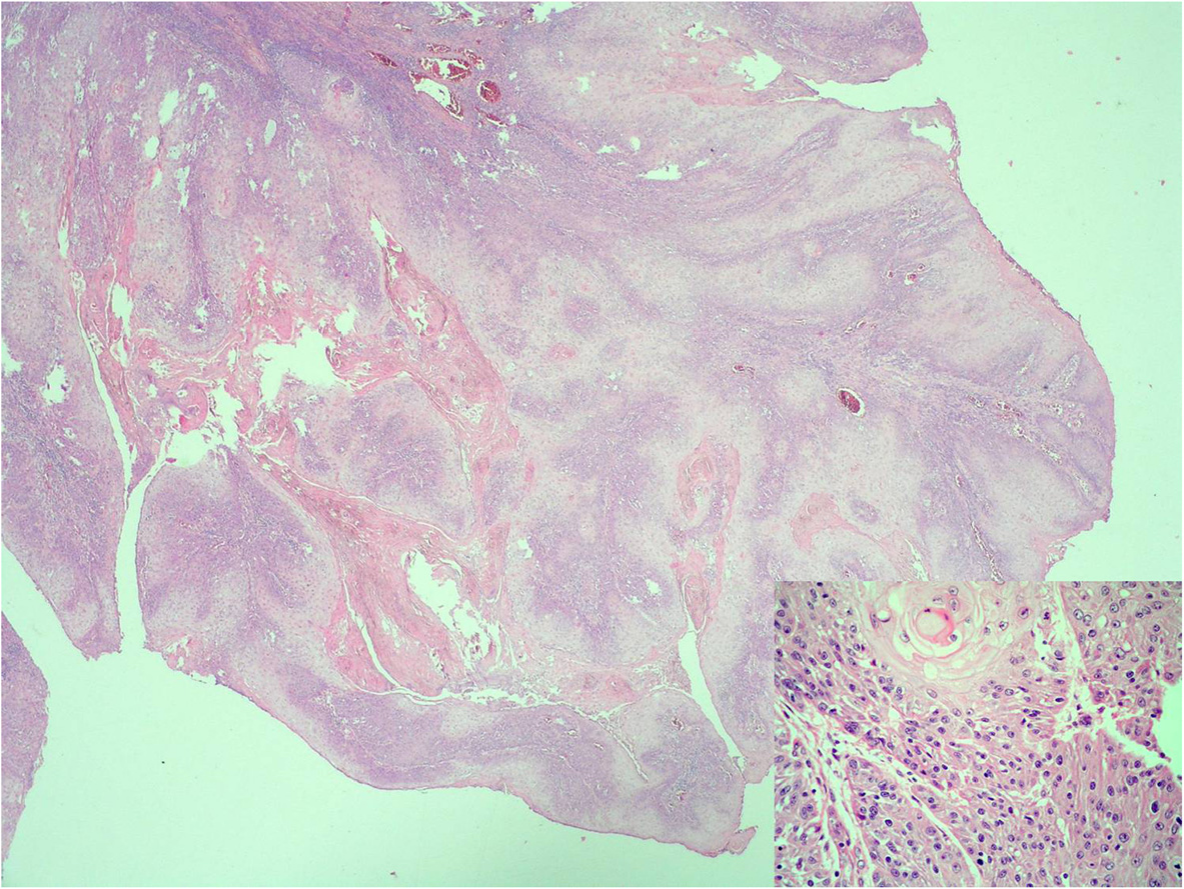
Primitive carcinoma pathological findings. Moderately differentiated squamous cell carcinoma of the penis showing invasion of the stroma and chronic inflammatory response (hematoxylin and eosin; original magnification 20×). *Inset*: keratinic pearl surrounded by pleomorphic epithelial cells with large eosinophilic granular cytoplasm and atypical nuclei with prominent nucleoli (hematoxylin and eosin; original magnification 400×)

A 49-year-old white man presented with a painful lump in his left breast. Three years before, he underwent a partial penectomy and inguinal radical lymphadenectomy, followed by adjuvant therapy (four cycles of cisplatin and 5-fluorouracil) for a penile invasive squamous carcinoma: pathological tumor stage 2, nodal stage 1 (1/16), and histopathological grade 2 (Fig. 1).

**Fig. 2:**
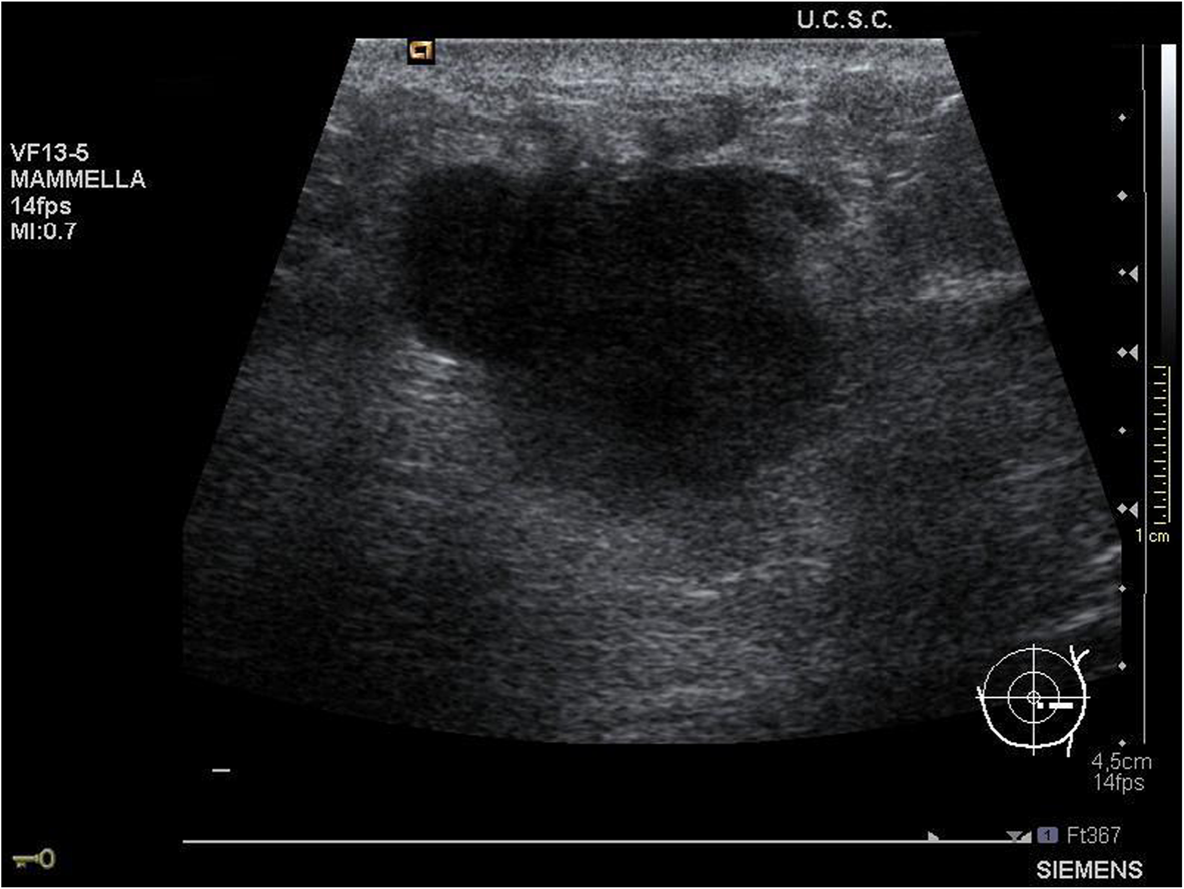
Radiological aspect. Breast ultrasonography showed a solid non-homogeneous hypoechoic lesion with partially regular contour

A clinical examination showed a 2 cm irregular-shaped firm lump, palpable near his left nipple. There were no palpable axillary lymph nodes and collaterally there were no signs of tumoral recurrence on his penile stump. Breast ultrasonography showed a 2 cm-sized irregular hypoechoic nodularity, without axillary lymph nodes involvement (Fig. 2).

A 25 G fine-needle aspiration was then performed on the breast lesion, and a cytologic examination determined the presence of a carcinoma. A full body computed tomography (CT) scan and bone scintigraphy were therefore performed, defining a metastatic disease: multiple pulmonary and bone metastases. Differentials of a secondary versus a malignant primary tumor were considered and in view of a diagnostic dilemma the breast lesion was excised.

**Fig. 3:**
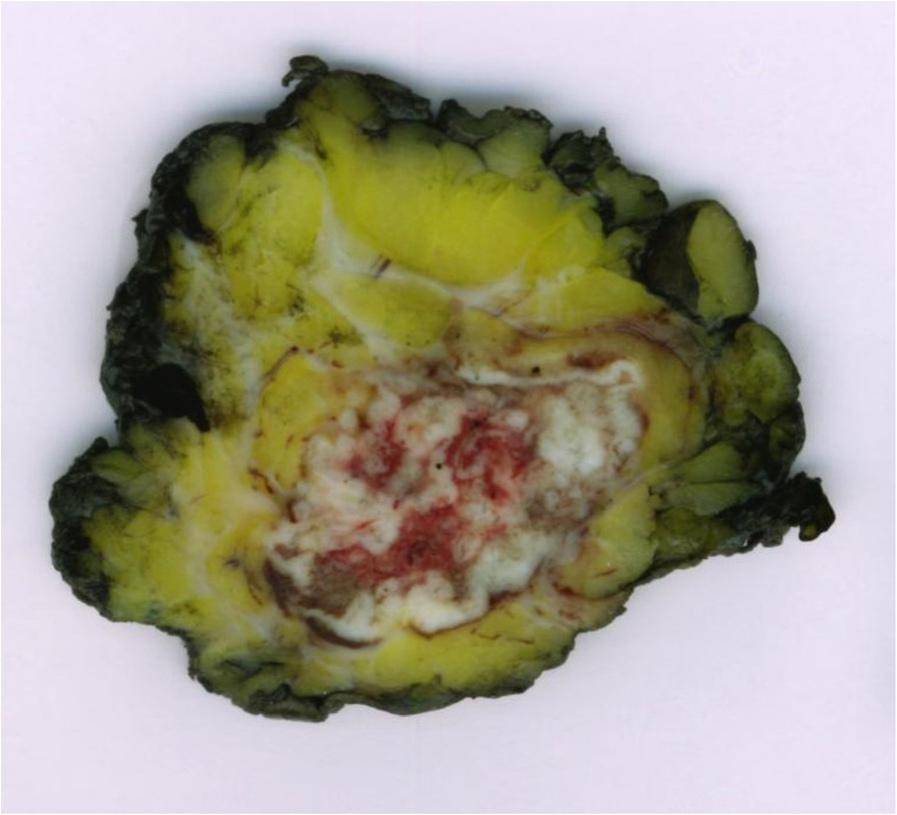
Surgical specimen. Macroscopic appearance of the breast metastasis

**Fig. 4:**
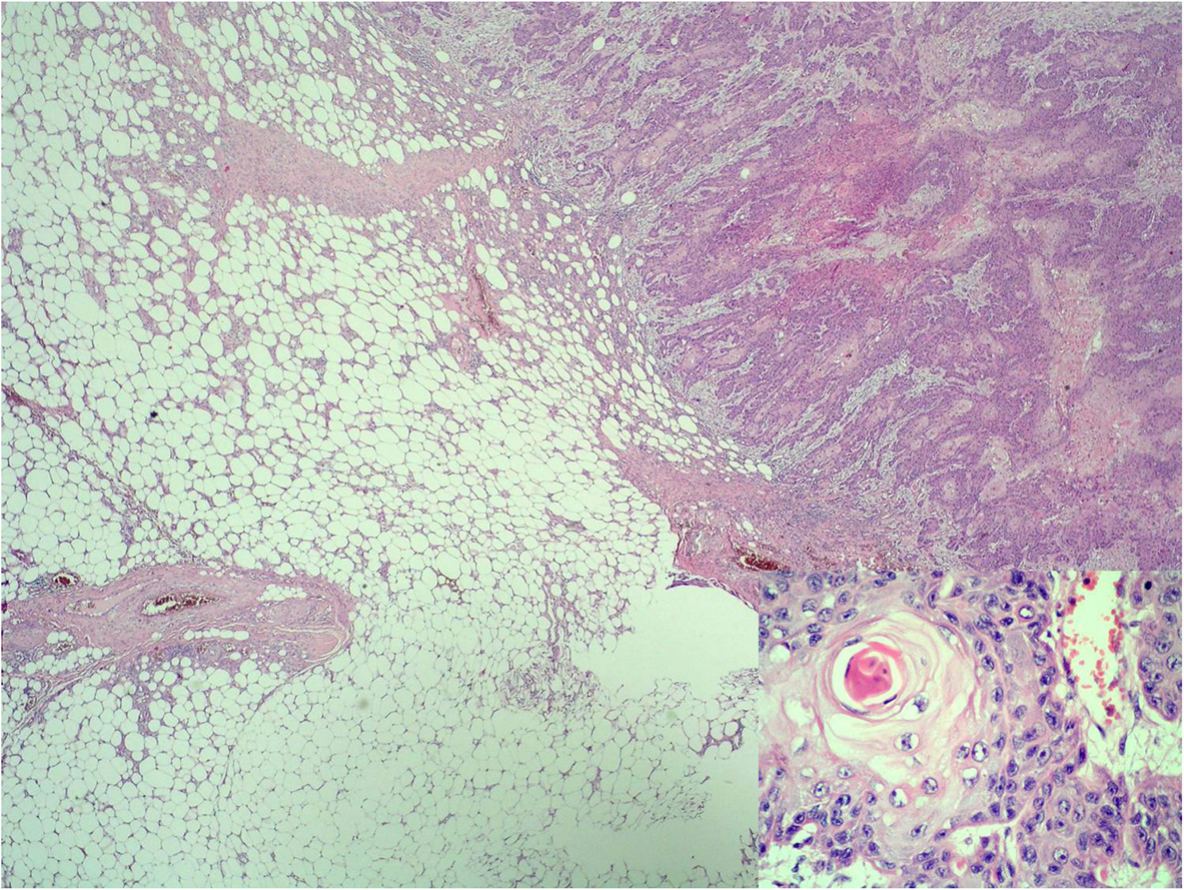
Breast metastasis pathological findings. Metastasis of squamous cell carcinoma in the breast parenchyma (hematoxylin and eosin; original magnification 20×). *Inset*: keratinic pearl surrounded by neoplastic epithelial cells with the same morphologic features of the primitive squamous cell carcinoma of the penis (hematoxylin and eosin; original magnification 400×)

Pathological examination revealed a 2 cm moderately differentiated invasive squamous cell carcinoma (Figs. 3 and 4); its histological features matched the previously resected penile cancer, so determining a penile relapsing disease that metastasized to the patient’s breast.

Following surgical treatment, the patient underwent a Taxol (paclitaxel) and gemcitabine-based chemotherapy regimen. Follow-up assessments, which were a total body tomography and positron emission tomography (PET)-CT scan, showed a 6 months’ disease stability, after which he developed a massive lung progression and died 8 months after surgery.

## Discussion

Penile squamous cell carcinoma is an uncommon malignancy, accounting for approximately 0.4 to 0.6 % of all cancer cases and 2 to 4 % of genitourinary neoplasms diagnosed among males in the USA and Europe [1]. It is considered a locoregional disease spreading locally and through lymphatic channels. Distant metastases with hematogenous spreading occur in advanced cases. The most common sites of metastatization are lungs, liver and bones [2–4]. There are some anecdotal reports of metastasis to the brain, dorsal spine, heart, retroperitoneum and skin described in the literature [5–10]; however, these cases are rare in clinical practice.

Breast metastases usually present as firm and well-circumscribed masses. These lesions are often mobile and superficial; they often involve the overlying skin [11]. On ultrasound, a metastasis usually appears as a well-circumscribed round or oval hypoechoic image [11, 12] and the pathological picture usually resembles extramammary primary cancer [12]. In our case, the breast lesion presented as a 2 cm, painful, irregular-shaped firm lump. Breast ultrasonography showed a round hypoechoic nodule with partially regular contour. Pathologic examination on the excised tissue determined its nature as a penile invasive squamous cell carcinoma metastasis.

The prognosis of patients with distant metastases of penile cancer is still very poor. Haas and colleagues reported complete remission in only five out of 40 patients with advanced penile cancer disease and none of these five patients had distant metastases. In the same series, only two of the patients with distant metastases showed a partial response and, in total, 36 of the 40 patients died [13]. The use of taxane-based regimens may improve on these poor results in the future. Pizzocaro and colleagues reported a partial response in two out of three patients with regional recurrence after inguinal lymphadenectomy [14].

Studies on second-line treatment are also needed in view of the often low response rate of first-line regimens.

## Conclusions

Penile carcinoma tends to have a predictable metastatic behavior, with predominant local progression or inguinal lymph node spreading. In cases of distant metastatization, the most common sites are lungs, liver and bones.

Here we describe the uncommon behavior of a penile squamous cell carcinoma that metastasized to the breast, presenting as a painful irregular-shaped firm lump. This case is unusual because of its site of metastatic presentation as well as its clinical pattern. Awareness of such a condition by physicians is mandatory in order to make an early diagnosis and start prompt and correct therapeutic planning.

## Consent

Written informed consent was obtained from the patient for publication of this case report and accompanying images. A copy of the written consent is available for review by the Editor-in-Chief of this journal.

## References

[1] Bleeker MC, Heideman DA, Snijders PJ, Horenblas S, Dillner J, Meijer CJ. Penile cancer: epidemiology, pathogenesis and prevention. World J Urol. 2009;27(2):141–50. 10.1007/s00345-008-0302-z 18607597

[2] Micali G, Nasca MR, Innocenzi D, Schwartz RA. Penile cancer. J Am Acad Dermatol. 2006;54:369–91. 10.1016/j.jaad.2005.05.007 16488287

[3] Culkin DJ, Beer TM. Advanced penile carcinoma. J Urol. 2003;170(2 Pt 1):359–65. 10.1097/01.ju.0000062829.43654.5e 12853775

[4] He Y, Markelov A, Amani M. Penile cancer metastases to the groin. Arch Clin Exp Surg. 2014;3(1):56–8.

[5] Lutterbach J, Pagenstecher A, Weyerbrock A, Schultze-Seemann W, Waller CF. Early-stage penile carcinoma metastasizing to brain: case report and literature review. Urology. 2005;66(2):432. 10.1016/j.urology.2005.02.011 16098372

[6] Moiyadi AV, Tongaonkar HB, Bakshi GK. Symptomatic intracranial metastasis in penile carcinoma. Indian J Urol. 2010;26(4):585–6. 10.4103/0970-1591.74471 PMC3034073 21369397

[7] Lal P, Halder S, Datta NR. Carcinoma of the penis metastasizing to the dorsal spine. A case report. Urol Int. 1999;62:249–51. 10.1159/000030407 10567894

[8] Swierz J, Poznański J, Stawarz B. Metastasis of penile cancer to the heart in a 20-year-old patient. Wiad Lek. 1992;45:314–6. 1462597

[9] Shaw BL, Menolasino III MJ. Metastatic penile squamous cell carcinoma to the retroperitoneum in a man with human papillomavirus type 45. J Am Osteopath Assoc. 2008;108(6):310–2. 18587080

[10] Khandpur S, Reddy BS, Kaur H. Multiple cutaneous metastases from carcinoma of the penis. J Dermatol. 2002;29:296–9. 10.1111/j.1346-8138.2002.tb00266.x 12081161

[11] Bartella L, Kaye J, Perry NM, Malhotra A, Evans D, Ryan D, et al. Metastases to the breast revisited: radiological-histopathological correlation. Clin Radiol. 2003;58(7):524–31. 10.1016/s0009-9260(03)00068-0 12834635

[12] Komorowski AL, Wysocki WM, Mitus J. Metastasis to the breast – a clinical challenge in outpatient. Acta Chir Belg. 2005;105(1):59–61. 15790204

[13] Haas GP, Blumenstein BA, Gagliano RG, Russell CA, Rivkin SE, Culkin DJ, et al. Cisplatin, methotrexate and bleomycin for the treatment of carcinoma of the penis: a Southwest Oncology Group study. J Urol. 1999;161:1823–5. 10332445

[14] Pizzocaro G, Nicolai N, Milani A. Taxanes in combination with cisplatin and fluorouracil for advanced penile cancer: preliminary results. Eur Urol. 2009;55:546–51. 10.1016/j.eururo.2008.07.014 18649992

